# Vulnerability of the Myelin‐Axon Interface Uncovered by Subcellular Proteomics and Imaging of Alzheimer's Human Brain

**DOI:** 10.1002/alz70855_100247

**Published:** 2025-12-23

**Authors:** Yifei Cai, Iguaracy Pinheiro‐de‐Sousa, Mykhaylo Slobodyanyuk, Fuyi Chen, Tram Huynh, Jean E. Kanyo, Peiyang Tang, Lukas A. Fuentes, Amber Braker, Rachel Welch, Huttner J. Anita, Lei Tong, Peng Yuan, TuKiet Lam, Evangelia Petsalaki, Jüri Reimand, Angus C Nairn, Jaime Grutzendler

**Affiliations:** ^1^ Yale University, New Haven, CT, USA; ^2^ EMBL‐EBI, Hinxton, Cambridgeshire, United Kingdom; ^3^ University of Toronto, Toronto, ON, Canada; ^4^ Yale University School of Medi, New Haven, CT, USA; ^5^ Yale University School of Medicine, New Heaven, CT, USA; ^6^ Yale Keck MSPR, New Haven, CT, USA; ^7^ Yale School of Medicine, New Haven, CT, USA; ^8^ Ontario Institute for Cancer Research, Toronto, ON, Canada; ^9^ Yale University School of Medicine, New Haven, CT, USA

## Abstract

**Background:**

Myelin ensheathment is essential for rapid axonal electrical conduction, metabolic support and neuronal plasticity. In Alzheimer's disease (AD), disruptions in both myelin and axonal structures occur; however, the underlying mechanisms remain poorly understood.

**Method:**

To investigate the molecular and cellular mechanisms of myelin‐axon disruption, we developed novel proximity labeling subcellular proteomics of the myelin‐axon interface in postmortem human brains and AD‐model mice. We developed new computational algorithm to uncover cell‐cell‐communication between myelin‐axon and their disruption in AD humans. We applied super‐resolution expansion microscopy and high‐resolution confocal imaging to AD human postmortem brains and AD‐model mice to reveal myelin‐axon disruption and pathological changes.

**Result:**

Using proximity labeling subcellular proteomics of the myelin‐axon interface in postmortem human brains and AD‐model mice, we discovered dysregulated signaling pathways and ligand‐receptor interactions, including those involved in β‐amyloid processing, axonal outgrowth and lipid metabolism. Expansion microscopy confirmed the subcellular expression of top proteomic hits and revealed β‐amyloid aggregation within internodal peri‐axonal space and paranodal/juxtaparanodal channels. Although no overt changes in myelin coverage were observed, we found reduced paranode density and aberrant myelination and paranode positioning around amyloid plaque‐associated dystrophic axons.

**Conclusion:**

These findings highlight the myelin‐axon interface as a critical site of protein aggregation and disrupted neuro‐glial communication in AD. The molecular architecture of the myelin‐axon interface uncovered in this study provides a foundation for future mechanistic investigations in health and disease.

